# “My bitterness is deeper than the ocean”: understanding internalized stigma from the perspectives of persons with schizophrenia and their family caregivers

**DOI:** 10.1186/s13033-018-0192-4

**Published:** 2018-04-03

**Authors:** Yin-Ling Irene Wong, Dexia Kong, Lufei Tu, Rosemary Frasso

**Affiliations:** 1 School of Social Policy & Practice, University of Pennsylvania, 3701 Locust Walk, Philadelphia, PA 19104-6214 USA; 2 College of Population Health, Thomas Jefferson University, 901 Walnut Street, 10th Floor, Philadelphia, PA 19107 USA

**Keywords:** Internalized stigma, Schizophrenia, Family caregiving, China

## Abstract

**Background:**

It is estimated that 8 million of the Chinese adult population had a diagnosis of schizophrenia. Stigma associated with mental illness, which is pervasive in the Chinese cultural context, impacts both persons with schizophrenia and their family caregivers. However, a review of the literature found a dearth of research that explored internalized stigma from the perspectives of both patients and their caregivers.

**Methods:**

We integrated data from standardized scales and narratives from semi-structured interviews obtained from eight family-dyads. Interview narratives about stigma were analyzed using directed content analysis and compared with responses from Chinese versions of the Internalized Stigma of Mental Illness Scale and Affiliated Stigma Scale. Scores from the two scales and number of text fragments were compared to identify consistency of responses using the two methods. Profiles from three family-dyads were analyzed to highlight the interactive aspect of stigma in a dyadic relationship.

**Results:**

Our analyses suggested that persons with schizophrenia and their caregivers both internalized negative valuation from their social networks and reduced engagement in the community. Participants with schizophrenia expressed a sense of shame and inferiority, spoke about being a burden to their family, and expressed self-disappointment as a result of having a psychiatric diagnosis. Caregivers expressed high level of emotional distress because of mental illness in the family. Family dyads varied in the extent that internalized stigma were experienced by patients and caregivers.

**Conclusions:**

Family plays a central role in caring for persons with mental illness in China. Given the increasingly community-based nature of mental health services delivery, understanding internalized stigma as a family unit is important to guide the development of cultural-informed treatments. This pilot study provides a method that can be used to collect data that take into consideration the cultural nuances of Chinese societies.

**Electronic supplementary material:**

The online version of this article (10.1186/s13033-018-0192-4) contains supplementary material, which is available to authorized users.

## Background

The pervasiveness of stigma associated with mental illness across societies with different socio-cultural background and economic development has been well documented in the research literature [1, 2]. As a complex social process, stigma occurs when human differences are labelled such that cognitive separation between “us” and “them” exists, and when cultural stereotypes are incorporated in the mindset by most members of a social group to the extent that minority members that are labelled experience significant status loss and reduced life opportunities [3, 4]. Stigma of mental illness is a multi-faceted phenomenon requiring an understanding from the perspectives of the general public, healthcare providers, persons with mental illness, and their family members [5].

Self-stigma occurs when individuals who bear a label of a discredited person direct the public’s stigmatized attitudes towards themselves. Self-stigma captures the adverse psychological processes that persons with mental illness experience because of negative stereotypes, prejudice, and discrimination they encounter in daily social interactions and upon the awareness that valuable societal resources such as employment, healthcare, and housing are denied or less accessible to them because of a psychiatric diagnosis. Self-stigma has been conceptualized as a three-stage process including the awareness, agreement and self-concurrence of negative stereotypes [3, 6]. A body of research has emerged documenting the adverse effects of self-stigma on self-esteem, empowerment, social inclusion, quality of life, help-seeking and treatment adherence among persons with psychiatric disabilities [7–9].

Associative stigma [10], also known as “courtesy” stigma [11], refers to the phenomenon that a person is devaluated or discriminated by virtue of association with a stigmatized individual [12, 13]. The prevalence of associative stigma in family caregivers has been documented in studies across different cultures [14–16]. Beliefs attributing to bad parenting skills as a trigger of mental illness and genetic models emphasizing biological vulnerability and inheritance, for example, are cited as potential factors explaining the severity of associative stigma in parents of persons with psychotic disorders [14, 17]. In parallel with self-stigma, affiliate stigma refers to the extent to which family caregivers internalize the prejudicial attitudes toward themselves, resulting in the use of negative affective, behavioral, and cognitive responses to cope with the spoiled identity as a close associate of someone with mental illness [5].

In this exploratory, pilot study, we examined stigma of mental illness from the perspectives of persons living with schizophrenia and their family caregivers in a suburban community in China. Given that China is a collectivist society with a family-centered culture, mental illness adversely affects not only individuals diagnosed with the disorder but also members of the family [18–20]. Although there is a growing body of empirical literature on stigma in China, most extant studies adopt a conceptualization of stigma without much consideration of Chinese cultural values and ethos and were conducted in major urban centers [21–23]. A recent qualitative study of Chinese immigrants diagnosed with psychotic spectrum disorders in New York City highlights the social-cultural significance of local social networks in understanding how disclosure of mental illness may damage a person’s participation in the community [24]. Maintaining a respectable standing as a family is a salient cultural norm because of the kin-based nature of social networks, particularly in rural and suburban China. In this study, we explored cultural issues which might shape the experiences of persons with schizophrenia and their family members.

An epidemiological study estimated that .8% (8 million) of the adult population in China had a diagnosis of schizophrenia; among these individuals, about 90% were classified as having a moderate to severe disability [25]. Understanding the experiences of family caregivers is important given that 90% of persons diagnosed with schizophrenia lived with their families in China, compared to 60% in Great Britain and 40% in the United States [23, 26, 27]. Unique Chinese cultural characteristics, such as concerns over loss of “face” and the social norm which expects the reciprocation of favors among network members, further support our approach for studying stigma of mental illness as a family unit [19].

A review of the literature, however, found a dearth of research that explored internalized stigma from the perspectives of both persons living with schizophrenia and their family caregivers. The limited qualitative inquiries conducted to date were mostly from European nations [28–30] and there was only one study examining both self-stigma and affiliate stigma in a Chinese metropolis using standardized measures [31]. The current study is unique in that we relied upon the voices of persons with schizophrenia and their family caregivers, a “hard to engage” population because of the pervasiveness of stigma regarding mental illness in China, to identify “what matters the most” in their experiences of living with schizophrenia [32]. The study site is also unique in that participants were recruited from a rural transitioning to suburban community, characterizing a massive urbanization process in contemporary China [33].

## Methods

### Study site, sample and procedure

The study was conducted in a suburban district in Shanghai, one of four directly-controlled municipalities in China. The 2010 Census of China registered a population of more than 60,000 in the study community, representing a threefold increase from a population of close to 20,000 at the time of the 2000 Census. In 2010, 51% of the population was urban, compared to 37% in 2000. Urbanization and population growth in the study community were attributed to the reclamation of farm lands to residential communities with high-rise housing and commercial complexes.

The study sample comprised persons with schizophrenia who were members of the local office of a national semi-governmental organization promoting the rights of persons with disabilities, and caregivers of these individuals. Local office staff identified potential participants from a list of members with psychiatric disability based on two criteria: first, prospective participants’ homes had to be close to public transportation routes; and second, prospective participants had the cognitive capacity as ascertained by the office staff to participate in an interview. Of all persons on the membership list (30 males and 30 females), 31 members (19 females and 12 males) were deemed eligible for further contact by local office staff. The membership list did not provide information about the psychiatric diagnoses of potential participants.

All potential participants were contacted by phone to explain the purpose of the study and to verify their interest in participation. Eligible participants were (1) a resident in the study community for 12 months or more; (2) at least 18 years old; (3) able to sign a written consent form and agree to participate in the study; and (4) designated to have a diagnosis of schizophrenia by self-report and report by a family member. To create index participant-family caregiver dyads, individuals meeting the above criteria were asked for permission to interview one of their family caregivers. We use acronyms, IP and FC, respectively, to indicate if a participant was the index participant (IP-persons with schizophrenia) or a family caregiver (FC) going forward. Eligible family caregivers were non-members in the national organization and assumed not to have a psychiatric disability. However, they did meet the first three aforementioned inclusion criteria. A final sample of 17 IP/FC dyads (55% of the 31 members identified by the local office staff) gave consent and completed a structured interview. Of the 14 non-participants, only two were refusals. Other reasons for non-participation included hospitalization, lack of competence to be interviewed as assessed by the research interviewers at the time of the interview, and failure of interviewers to contact potential participants after multiple attempts.

During the structured interviews, the IPs was assessed in regard to their ability to complete a follow-up qualitative interview. Twelve of the 17 IP/FC dyads were invited to participate in a semi-structured interview with a list of open-ended questions. Eight dyads agreed to participate and 4 refused. Semi-structured interviews were conducted 6–16 days after the structured interviews.

All procedures performed in this study were in compliance with the ethical standards as laid out in the 1964 Helsinki declaration and its later amendments. Ethics approval was obtained from the University of Pennsylvania Office of Regulatory Affairs Institutional Review Board (Federalwide Assurance # 00004028). All study participants provided their written informed consent at both phases of the study.

### Data collection

The study focused on internalized stigma in persons with schizophrenia and their family caregivers. Data collection procedures for the structured interview and semi-structured interview are detailed below.

#### Structured interviews

All structured interviews were conducted by two teams of interviewers who worked in pairs. A week-long intensive workshop was conducted to train the interviewers using small group discussions and role playing. The structured interview protocol was piloted with four members of the national organization and their four family caregivers in another district of Shanghai to ensure that the question items were culturally valid and linguistically appropriate. Feedback from these respondents was incorporated in the final version of the structured interview protocol.

Basic demographic characteristics, including age, gender, educational attainment, and marital status, were collected from the IP/FC dyads. In addition, clinical characteristics, including psychiatric diagnoses, history of mental health treatment, and psychiatric medication taken, were collected from participants with schizophrenia. Interviews for IP and FC were conducted separately.

Self-stigma was measured with a Chinese version of the Internalized Stigma of Mental Illness (ISMI) scale. The ISMI was originally developed with a sample of 127 outpatients of mental health services at a Veteran Affairs medical center in the United States [34]. The scale was translated into Chinese and field-tested with 206 patients by Li and colleagues in a psychiatric department providing inpatient and out-patient care in a general hospital in China [35]. The ISMI (Chinese version) demonstrated sound psychometric properties, including high internal consistency reliability, good construct validity, and reasonable discriminant validity. It replicated the factor structure of the ISMI in the original study [34]. The ISMI is composed of 22 items with 4 domains, namely, alienation, perceived discrimination, social withdrawal, and stereotype endorsement. Study participants rated the extent to which they endorsed each item on a Likert scale ranging from [1] strongly disagree to [4] strongly agree. A higher score indicated a higher level of self-stigma. The Cronbach’s alpha of the scale is .86 for the study sample (Additional file 1).

A 22-item Affiliate Stigma Scale (ASS), developed by Mak and colleague, and written in Chinese, was used to measure internalized stigma experienced by individuals who are closely affiliated with persons with disabilities [5]. Represented by three domains, namely, affective, behavioral, and cognitive, the ASS was tested with 210 family caregivers of persons with intellectual disability and 108 family caregivers of persons with psychiatric disability in a metropolitan area in southern China. The ASS has the same response set as the ISMI. An examination of the psychometric properties of the scale suggested excellent internal consistency and good predictive validity on subjective burden [5, 36]. The alpha for the scale is .96 for family caregivers in the current study.

#### Semi-structured qualitative interviews

An interview guide with a list of open-ended questions was constructed to facilitate the discussion of participants’ experience of living with schizophrenia (IP) or caring for a family member who was diagnosed with schizophrenia (FC). The interviews began with asking both IP and FC to share an account of the onset of schizophrenia and the things they did to cope with the situation. The interviewers then asked the participants to talk about reaction of other people in the community towards them. The interviews concluded with additional thoughts that the participants had in regard to living in the community. In each section of the interview, the interviewers invited the participants to give examples to illustrate their thoughts and feelings.

Two bilingual (Chinese/Putonghua and English) research staff with graduate level education and training in qualitative data collection conducted the interviews. They worked in pairs; one interviewed the IP while the other interviewed the FC, separately. After each interview, the interviewers wrote a detailed memo to record salient observations and important remarks made by the study participants. Debriefing on the interviews was conducted in consultation with the senior author who is bilingual and had conducted qualitative research studies with persons with serious mental illness in the United States. Interviews lasted an average of 49 min for IP (ranging from 23 to 60 min) and 52 min for FC (ranging from 38 to 60 min).

All interviews were audio-recorded and transcribed verbatim by two graduate-level research assistants who were fluent in Putonghua (the official language in China) and the local dialect. These research assistants were not involved in the data collection phase of the project. Six transcripts were randomly selected and audited by the second author to assure the accuracy of the transcription.

### Data analysis

Data from standardized scales and semi-structured qualitative interviews were integrated to achieve a nuanced and contextualized understanding of stigma while retaining the integrity of each method, as recommended by Moran-Ellis and colleagues [37]. Directed content analysis, which is both a deductive and inductive approach to qualitative data analysis, was used in this study [38]. Daly argued that the combination of inductive and deductive reasoning can paint a fuller picture of an experience and be useful when expanding understanding of a phenomena is an important goal of the study [39]. Furthermore, when prior research or current theories have not been employed in context, are incomplete, or would benefit from further description, directed content analysis is a recommended method by allowing the researcher “to validate or extend conceptually a theoretical framework or theory” [38]. We adopted the directed content analysis approach because our goal was to expand understanding of stigma of mental illness in Chinese society, noting that prior work has fallen short of exploring the experiences of both the people living with schizophrenia and their family caregivers within the Chinese cultural context. By cross-validating responses to standardized scales with narratives articulated in semi-structured interviews, this method enables us to examine concordances and discrepancies of information procured by the two data collection methods, which in turn shed light on the lived experiences of stigma, discrimination and devaluation.

The procedures we followed for directed content analysis are detailed here. The first and second authors reviewed the ISMI and ASS, taking note of the conceptualization of the domains comprising the scale and the meaning of each scale item. We then independently reviewed all eight transcripts from the semi-structured interviews with IPs and highlighted relevant text fragments that reflected the meaning of items in the ISMI. When a text fragment was matched to a scale item in ISMI, the researchers referenced the detailed memo written by interviewer to contextualize the meaning of the text fragment. We also took notes of the story lines and events described in the semi-structured interviews to ensure that the text fragments identified truly matched the scale items. The same process was repeated for all 8 FC-transcripts and the ASS.

The researchers then organized the results in a spreadsheet with identified text fragments per each scale item placed in adjacent columns. A bilingual graduate-level research assistant who was not involved in the project was recruited to form a three-member team to identify and resolve discrepancies by discussion. Two tables showing the number of participants endorsing each scale item and number of text fragments found in the semi-structured interviews that matched each item of ISMI and ASS, respectively, were constructed to provide an overall distribution of the quantitative and qualitative findings. The text fragments were analyzed in the language they were collected (Chinese) to preserve cultural and linguistic nuances. Representative quotations that were chosen for dissemination in this manuscript were translated into English and back-translated to assure accuracy.

Comparing standardized scale scores and text fragments per study participant provides additional richness to the understanding of internalized stigma. A bar chart and a scatterplot were generated to show if IPs who scored high on the ISMI also provided high number of narratives and vice versa. The same approach was used to compare the response pattern in FCs.

To examine the interactive aspects of stigma in a dyadic relationship, a clustered bar-chart was constructed displaying the internalized stigma scores of the eight family-dyads. Three profiles, one dyad with convergent stigma scores (similar levels of stigma for both IP and FC) and two family-dyads with divergent stigma scores (one with high level for IP but low level for FC, and the other with low level for IP but high level for FC), were described in the results section to give nuances about internalized stigma in a dyadic relationship.

## Results

### Sample characteristics—IP/FC dyads

Of the 8 family-dyads (IP/FC), 7 were child-parent dyads (3 daughter/father, 3 son/mother, 1 daughter/mother) and 1 was a wife/husband dyad. IPs were, on average, 25 years younger than their caregivers, reflecting the relationship status of the dyads. Most IPs had a middle school education, whereas most FCs had primary school education or below. Most FCs were currently married, whereas only 2 female IPs were married at the time of data collection. All 3 male IPs had never married.

The age when IPs first saw a mental health professional for treatment of schizophrenia ranged from 15 to 40 years old, with an average age of 24. The duration since a formal diagnosis of schizophrenia ranged from 2 to 48 years, with 6 of the 8 IPs being diagnosed for 12–20 years. In addition, 6 IPs experienced at least one inpatient psychiatric hospitalization during their lifetime with an average age of 22 years old at the time of first hospitalization. Although all IPs were taking psychiatric medication at the time of data collection, only 3 were currently treated by a physician for psychiatric issues.

In the first two sub-sections below, we present summary data according to the domains of the two stigma scales. The eight dyads are identified by a letter (A to H).

### Stigma scores and narratives among IPs (persons with schizophrenia)

A total of 68 text fragments matched the scale items in the ISMI: 16 in the alienation domain; 30 in the perceived discrimination domain; 21 in the social withdrawal domain; and 1 in the stereotype endorsement domain. Table 1 presents the number of participants (out of a total of 8) who agreed with each scale item of the ISMI (column 1) and the number of text fragments matched per scale item (column 2). Within each domain, the items were ranked from the highest to the lowest number of participants endorsing (i.e., rated “agree” or “strongly agree”) the item.

**Table 1:** Internalized stigma of mental illness: number of participants agreed and number of text fragments matched

Statement/item	# agreed	# of text fragments
Alienation		
1. Because of mental illness, I became the burden of my family	6	4
2. Having a mental illness has spoiled my life	6	3
3. I feel inferior to others who don’t have a mental illness	4	6
4. People without mental illness could not possibly understand me	4	0
5. I am disappointed in myself for having a mental illness	3	2
6. I feel out of place in the world because I have a mental illness	2	1
7. I hate myself because I have mental illness	2	0
Perceived discrimination		
1. People discriminate against me because I have a mental illness	4	18
2. Others think that I can’t achieve much in life because I have a mental illness	4	2
3. Nobody would be interested in getting close to me because I have a mental illness	3	5
4. People ignore me or take me less seriously just because I have a mental illness	2	5
Social withdrawal		
1. I don’t talk about myself much because I don’t want to burden others with my mental illness	5	1
2. I don’t socialize as much as I used to because my mental illness might make me look or behave “weird”	4	8
3. Negative stereotypes about mental illness keep me isolated from the “normal” world	4	0
4. I’m afraid to talk about mental illness and related topics	3	0
5. I stay away from social situations in order to protect my family or friends from embarrassment	3	0
6. Because of mental illness, I often want to avoid familiar people and environment	2	8
7. I avoid getting close to people who don’t have a mental illness to avoid rejection	2	0
8. Being around people who don’t have a mental illness makes me feel out of place or inadequate	1	4
Stereotype endorsement		
1. Because I have a mental illness, I need others to make most decisions for me	5	1
2. I can’t contribute anything to society because I have a mental illness	4	0
3. People with mental illness cannot live a good, rewarding life	3	0

#### Alienation domain

Alienation captures the experience of internalizing a “spoiled identity” given one’s membership in a discredited minority group [11, 34]. The two most frequently endorsed statements, perceiving self as a family burden and feeling that mental illness has spoiled one’s life, had a total of 7 matched text segments in the transcripts. A narrative exemplifying how having mental illness has spoiled an IP’s life is given in the following:

“The impact is that I very much regretted in my entire life that I wasn’t able to take Zhong Kao (i.e., the senior high school entrance examination). If I could have taken the exam, I would pursue Chinese medicine. I felt it was the greatest dream in my life but I gave it up, quiet a pity.”

In addition, six text fragments were found matching the scale item “feeling inferior to others who don’t have a mental illness.” IPs spoke about having an inferiority complex, having no self-confidence at all, daring not to talk back when other people verbally attacked them, and daring not to face other people. IPs also expressed total dissatisfaction with everything and giving up on oneself, as well as feeling out of place in the world by expressing that they were different from normal people as “a disabled person.”

#### Perceived discrimination domain

The majority of text fragments in this domain matched the statement “people discriminated against me because I have a mental illness.” IPs talked about the injustice and maltreatment they endured at the hands of their employers or coworkers who knew about their illness. They gave examples about being given the “worst” job assignments by their supervisors, being fired because other people spoke badly about them to their boss, and having had work tools stolen by their coworkers. Another important source of discrimination was from the illness itself, including having to take psychiatric medication for life, forced to enter a psychiatric facility without their consent, and having a mother who was also diagnosed with schizophrenia. One study participant remarked impassionedly about the pervasiveness of discrimination she experienced by describing in detail about how “bad” people in her village provoked, bullied, and maltreated her, even after they all knew she had mental illness.

“Everywhere in the workplace and society, people did not let me get through. Everywhere I felt suffocated. People starred at me and bullied me.”

IPs also contributed narratives that described experiences of marginalization and devaluation, specifically mentioning concerns that (1) others think that they cannot achieve much in life; (2) nobody would be interested in getting close to or staying connected with people with mental illness; and (3) others ignore or take them less seriously because of mental illness.

“They [neighbors] thought that I could not do anything. When they saw that I cooked dinner, made snacks, or whatever, they would say: ‘Look! I can’t believe she also could do that.’ I thought I must look different than others.”

“There was a girl who worked at the same factory as mine, but we are not connecting now. I thought we were once very close…but when she found out I got this illness, she contacted me no more.”

#### Social withdrawal domain

Scale items in the social withdrawal domain were connected to behavioral responses adopted by IPs, such as reducing, avoiding and withdrawing from interpersonal contacts. While there are eight scale items in this domain, only four items, including being a burden to others, not socializing because of looking or behaving “weird,” avoiding familiar people and environments, and feeling out of place when being around people, have matched text fragments in the transcripts.

IPs spoke about not engaging socially because of the fear of being a burden to other people. They were aware of their difference from others and concerned about disclosing their mental illness.

“Other people would be worried. So I don’t want to let them know. I’ll take good care of myself. When I recover from the illness, then I may talk with them.”

#### Stereotype endorsement domain

Although there was a moderate level of endorsement on scale items comprising the stereotype endorsement domain, only one matched text fragment was found in the eight interview transcripts. Speaking about the first time she consulted a doctor at a young age after experiencing symptoms of delusion and hallucination, an IP lamented for not being able to choose whether or not to take psychiatric medication.

“I don’t have the right to choose. They make decisions for me. They are my family. It’s not some strangers who ask me to take psychiatric medication.”

This IP went on to explain how being diagnosed with schizophrenia had negatively affected her life, including decimating her chance of getting married.

### Stigma scores and narratives among FCs (family caregivers)

A total of 87 text fragments in the FCs’ transcripts matched the scale items of the ASS, with 47, 21, and 19 fragments in the affective, behavioral, and cognitive components of the scale, respectively.

#### Affective domain

This domain captures the heightened emotions that FCs experienced as close relations of persons living with schizophrenia [5]. As Table 2 shows, all but one item in this domain were endorsed by a majority of FCs. The top three scale items with the most matched text fragments were: (1) sense of helplessness; (2) feeling emotionally disturbed; and (3) being under great pressure because of having an ill family member. Exemplary narratives of feeling distressed and burdened include:

**Table 2:** Affiliate Stigma Scale: number of participants agreed and number of text fragments matched

Statement/item	# agreed	# of text fragments
Affective		
1. I feel emotionally disturbed because I have a family member with mental illness	7	11
2. The behavior of a family member with mental illness makes me feel embarrassed	7	2
3. I feel inferior because I have a family member with mental illness	7	2
4. I feel that I am under great pressure because I have a family member with mental illness	6	10
5. I feel sad because I have a family member with mental illness	5	8
6. I feel helpless for having a family member with mental illness	5	14
7. I worry that other people would know I have a family member with mental illness	3	0
Behavioral		
1. I dare not tell others that I have a family member with mental illness	6	10
2. When I am with a family member having mental illness, I would keep a relatively low profile	5	0
3. I dare not to participate in activities related to mental illness lest other people would suspect I have a family member with mental illness	5	0
4. Given that I have a family member with mental illness, I reduce contact with my friends and relatives	4	2
5. I reduce going out with a family member with mental illness	3	0
6. Given that I have a family member with mental illness, I reduce contact with the neighbors	2	9
7. I reduce interacting with a family member with mental illness	1	0
8. I avoid communicating with a family member having mental illness	1	0
Cognitive		
1. Having a family member with mental illness imposes a negative impact on me	7	2
2. My reputation is damaged because I have a family member with mental illness	6	0
3. Having a family member with mental illness makes me think that I am incompetent compared to other people	4	2
4. Having a family member with mental illness makes me think that I am lesser to others	4	2
5. Other people would discriminate against me if I am with a family member having mental illness	3	10
6. Having a family member with mental illness makes me lose face	3	2
7. People’s attitude towards me turns bad when I am together with a family member having mental illness	3	1

“(It is) way too bitter! My bitterness is too hard to tell. I feel my bitterness is deeper than the ocean.”

“He (my son) got this illness and my mind was burdened heavily…What if he won’t get better…My heart was so full of worries. Because of him, I got heart disease.”

Feelings of sadness were expressed by mostly female FCs, including “my heart ached to the point of death,” “my heartache was simmering to the point that I wanted to cry my eyes out; but there was no place for my tears,” and “I cried until others thought I was going crazy; indeed, I was nearly crazy.” FCs also described the circumstances under which they were overwhelmed with sadness, including witnessing their ill family member being openly shunned by their extended family, and when their acquaintances repeatedly enquired about their ill relative.

Feeling embarrassed because of the “bizarre” behaviors of an ill family member and harboring a sense of inferiority were noted in the interview transcripts. For example, one male FC spoke about the humiliation he endured when his wife, during a psychotic episode, paid repeated visits to his workplace. Another FC reflected on how her self-image gradually changed after her son was diagnosed with schizophrenia.

“People in my factory didn’t know about my family’s situation. When she came to the factory, then everyone knew…I felt so deeply bothered by her being there.”

“Before I had a competitive disposition (I considered myself to be stronger than others). Now I always feel I am lesser than others, being inferior.”

#### Behavioral domain

The behavioral domain elicited actions that FCs took to cope with the blame and shame associated with having an ill family member. As the second panel in Table 2 shows, text fragments were found in 3 of 8 scale items comprising this domain. Items without any matched text fragments included keeping a low profile, reducing going out with the IP, daring not to participate in activities related to mental illness, reducing interactions with the IP, and avoiding communication with the IP.

The item “I dare not tell others that I have a family member with mental illness” was endorsed by 6 of the 8 FCs. A review of the contexts in which matched narratives occurred suggests that this behavioral response was used when FCs considered their family members to be not seriously ill and could pass as a “normal” person. For example, parents of IPs who were considered to have a higher level of functioning, including the ability to hold a job, talked about keeping the illness a family secret.

“I don’t want, I don’t want my neighbors to know. My thought is that wherever we live, we wanted to keep good relationships with our neighbors.”

“I’m certain about that…I definitely don’t want to mention anything about my daughter…Keep her illness a secret…It’s definitely a little better for my daughter. The fewer people know about it, the better.”

Other FCs spoke about reducing contact with neighbors, friends and relatives. For example, an aging mother of a mentally ill son was able to avoid contacting neighbors after she moved from her village home to an apartment in a high-rise housing complex because of land acquisition.

“I don’t go out and interact with others. What’s the point of going out? I don’t have contact with people here. I avoid them. I don’t know anyone in this neighborhood.”

#### Cognitive domain

The extent to which FCs perceived their social standing dwindle in the eyes of other people constitutes the cognitive aspect of affiliate stigma. As the third panel of Table 2 showed, FCs endorsed, to a varying degree, statements in ASS which elicited a range of negative consequences because of having a close relative with schizophrenia.

Most of the text fragments identified in this domain spoke to the discriminatory treatments experienced by FCs. Circumstances in which discrimination took place included an employer who used deception to obtain a FC’s signature to terminate her son’s hiring contract, a relative who stopped inviting a FC to social outings out of concern over compulsive-eating of FC’s son, and a co-worker who distanced herself from a FC after learning that she had a son with schizophrenia. Other expressions of “discrimination by association” pointed to the “contamination” of the whole family because one member was diagnosed with MI.

“People know that my son has mental illness. They would look at him eerily because of his illness, right? They would say, ‘Oh my God, how come this family has mental illness?’”

“I always consider my family to be different from an ‘ordinary’ family. Certainly consider that my family would be discriminated by others, being looked down upon. This is our basic psychology. Families that have mental illness almost always think like this.”

In addition, text fragments identified in the transcripts illustrated FCs’ own tarnished view because of the blood relationship they had with a relative with schizophrenia. Even in the absence of overt discrimination by other community members, FCs carried the burden of shame and perceived them as belonging to a lower social order.

“I always go out. If people mention it (anything about mental illness in my family), what they say is not with a bad intention. But for me, this is a very shameful thing to bear.”

### Comparison between number of text fragments and scores on standardized scales

**Fig. 1:**
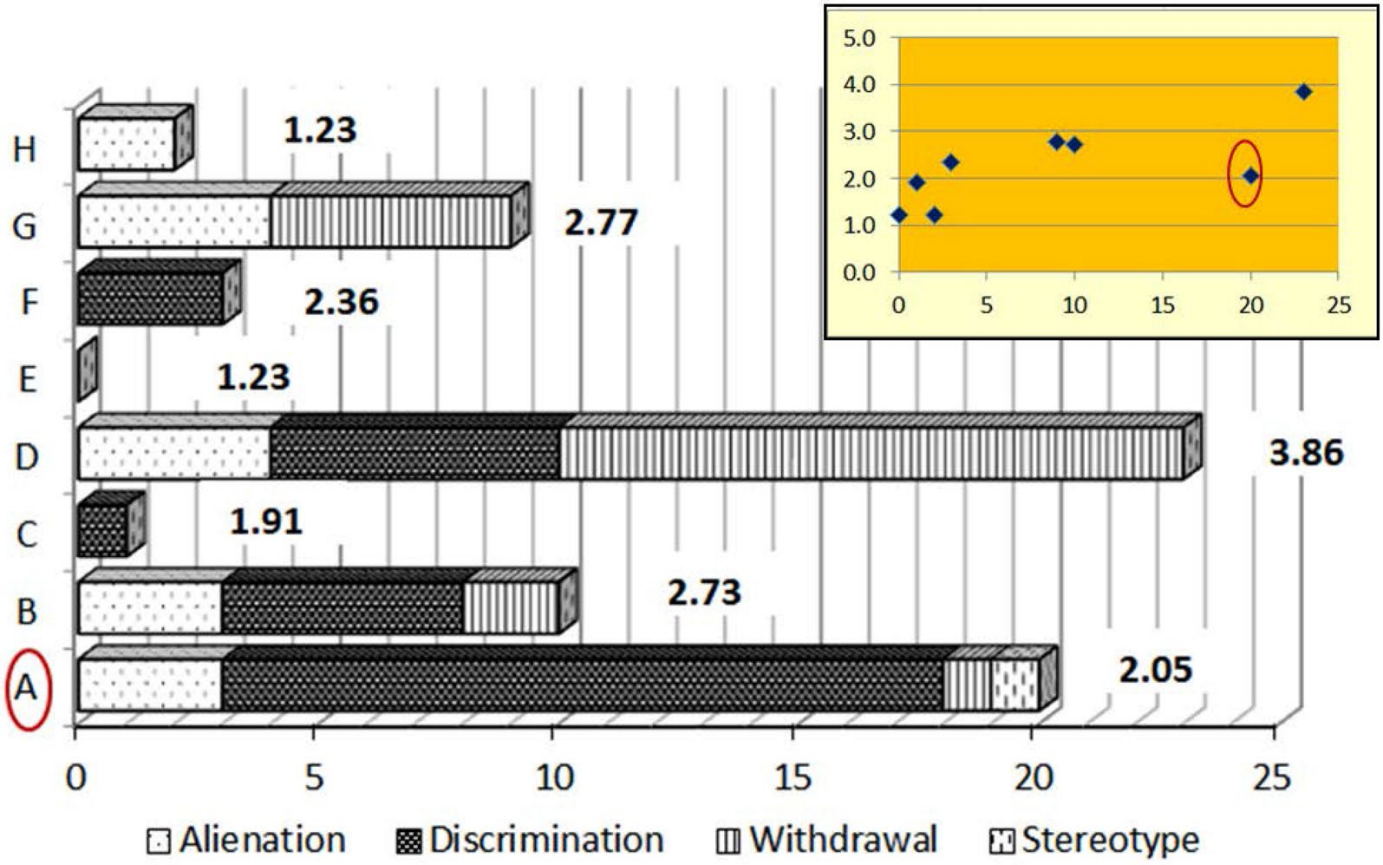
Comparison between number of text fragments and ISMI scores for IPs

**Fig. 2:**
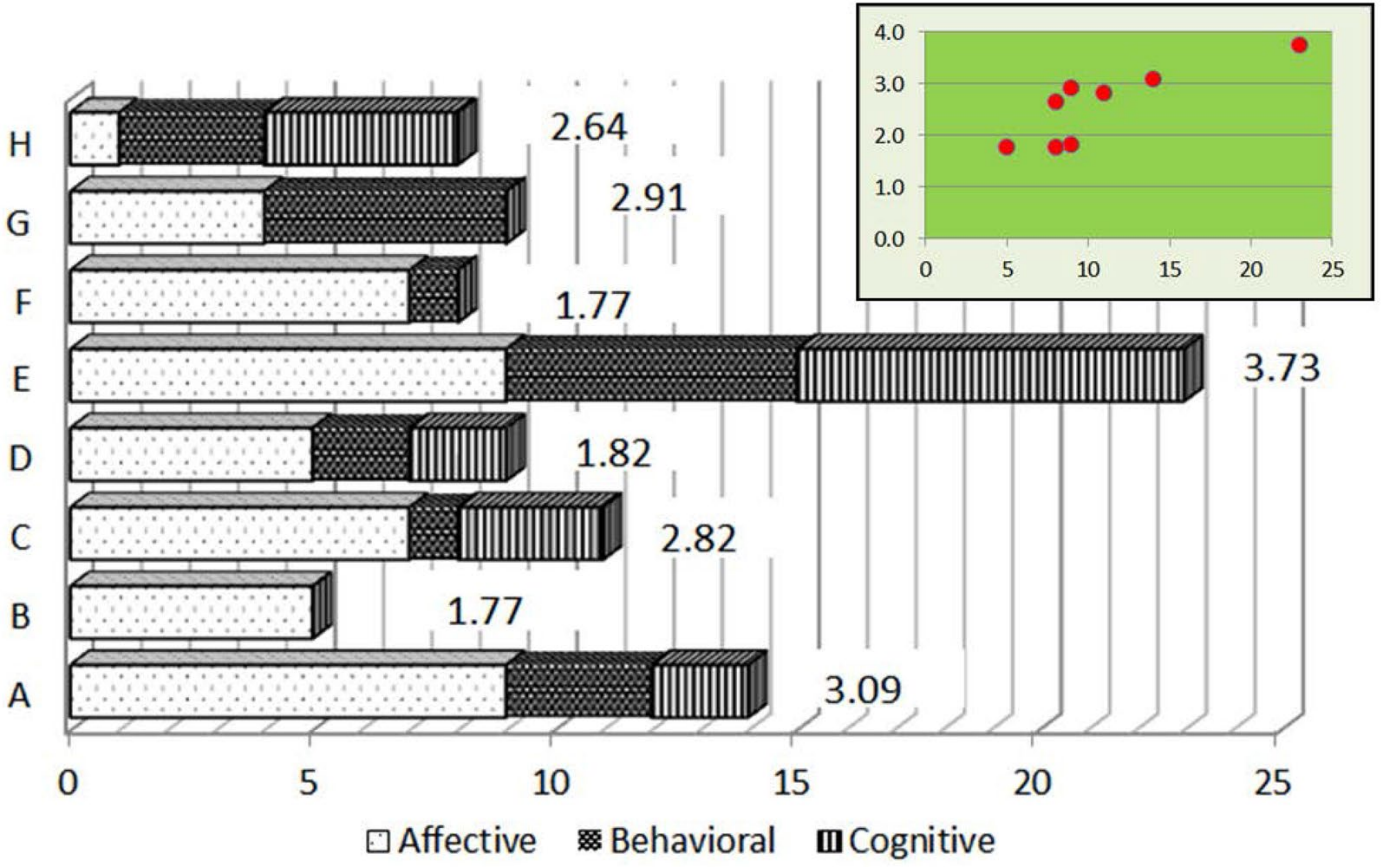
Comparison between number of text fragments and ASS scores for FCs

The bars in Figs. 1 and 2 show the number of text fragments per study participant (A to H) identified in the semi-structured interviews that matched items in the domains of the ISMI and ASS, respectively. The standardized scale scores of participants are placed beside the bars. The scatterplots inserted at the upper right corner of the two charts display graphically the relationship between number of text fragments (on x-axis) and standardized scale scores (on y-axis). Among the 8 IPs, those who provided high number of text fragments also scored high on the ISMI with one exception (see Fig. 1). While IP-A scored fourth in rank among 8 IPs from the lowest to highest ISMI score, 20 text fragments were identified in IP-A’s semi-structured interview that matched the ISMI items (number of text fragments found in IP-A are the second highest, next to IP-D, who also scored the highest on ISMI). Of the 20 text fragments contributed by IP-A, 15 matched the perceived discrimination domain. IP-A spoke profusely during the semi-structured interview about numerous occasions in which neighbors, family members and coworkers discriminated against her because they knew she had a psychiatric diagnosis. It is important to note that many of the occasions she spoke about dated back to her prior residence in her village 7 years ago, before she moved to the housing complex where she was interviewed.

Figure 2 suggests that the extent of endorsement to the scale items in ASS is consistent with the number of text fragments identified in the semi-structured interviews for family caregivers. This is reflected in the scatterplot, as there is no outlier among FCs, which was in the case of IPs.

### Lived experience of stigma in a dyadic relationship

**Fig. 3:**
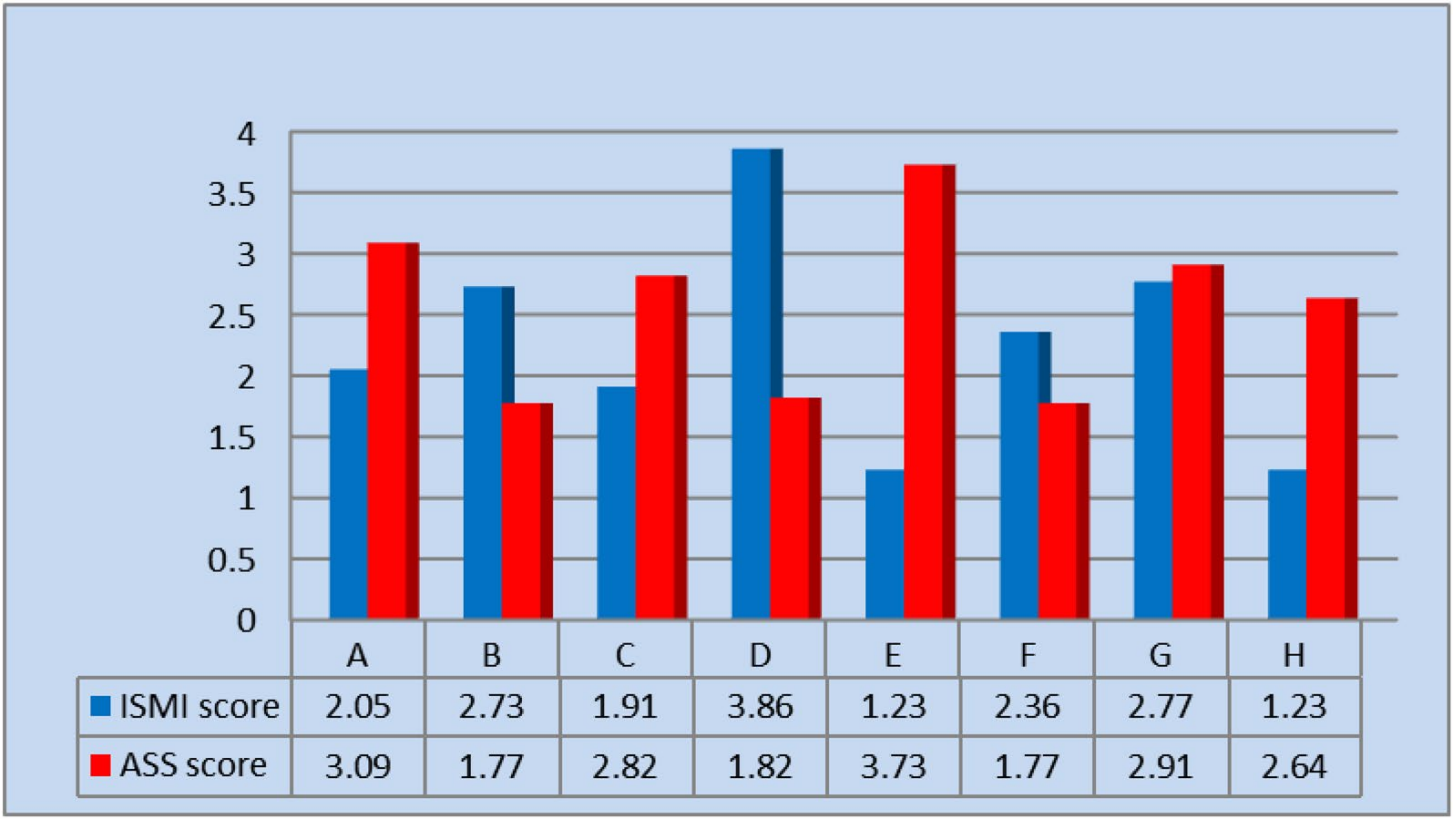
Internalized stigma score of the study sample by family dyads

Figure 3 is a clustered bar-chart showing the stigma scale scores of the 8 family-dyads. The mean ISMI and ASS scores of the study sample were 2.27 and 2.57, respectively. The IP and FC of dyad G reported similar scores on their respective stigma scales. The IP of dyad D scored the highest on ISMI, whereas the FC scored second lowest on ASS. Conversely, the IP of dyad E reported one of the two lowest ISMI scores, whereas the FC scored the highest on ASS. We focus on these three contrasting dyads (D, E, and G) in illustrating the interactive aspects of stigma in a dyadic relationship.

Dyads D and E were similar in a number of demographic and clinical characteristics. They were both son–mother dyads with IP-D and IP-E aged 33 and 35, respectively, and FC-D and FC-E aged 55 and 60, respectively. In regard to family structure, both IPs were never married, were the only biological child of their parents, and were co-residing with them at the time of the study. Both IPs were first hospitalized for mental illness at age 19.

IP-D experienced multiple psychiatric hospitalizations (7–8 times), which he described as involuntarily commitments. He abominated about being corporally punished by his mother as a child and complained that his parents could neither reason with him nor respect him as a person. Consistent with the high ISMI score reported (3.86 out of a possible 4) by IF-D, there were 23 text fragments identified in the semi-structured interview which matched the scale items of ISMI. A majority of these text fragments [13] was in the domain of social withdrawal (refer to Fig. 1).

“I just don’t go out, I go out as little as possible. Especially for people I know, I do not get in touch with them. For those whom I do not know, I am more willing to get a little closer.”

“It is true that I am…not getting along well with others. I’m just not good at socializing. Other people may be laughing and joking, very lively. But once I joined them, they would all stop talking. Then it became very awkward.”

FC-D admitted to have “forced” IP-D to seek psychiatric help after he was first diagnosed with depression and then with schizophrenia. While she had been keeping things to herself (i.e., not disclosing mental illness in her family to others) and did not associate with her neighbors and coworkers, she attributed her lack of social engagement to being an introvert, rather than to fear of discrimination. Although FC-D used such words as “no way to go” and “helpless… like no direction” to describe the distress she endured, she emphasized that religious faith provided her with tremendous support as a positive coping strategy.

“Now I do not have any pressure. Now I believe in God, not a bit of pressure… In the past, the pressure is great. I had always thought: how could I face this?”

The relatively few narratives extracted from FC-D’s semi-structured interview that matched ASS is a sharp contrast to the expression of sadness, behavioral avoidance, and sense of failure by FC-E, who reported a score of 3.73. There were 23 text fragments which matched items of ASS, including 9 in the affective domain, 6 in the behavioral domain, and 8 in the cognitive domain. One example for each domain is given below.

“Whenever he (my son) got decompensated, I was always thinking if there was anyone who could help me…I wanted to get him hospitalized. I wanted to get help from others, but I felt I was not able to do anything…I felt utterly helpless.” (Affective)

“I brought my son to this world and he got this illness. I always feel ill-at-ease when interacting with other people…Before his illness I was very cheerful and outgoing…I am not willing to make friends anymore.” (Behavioral)

“Out there in society, I always consider myself to be shorter than others by one-third. Hmm, I used to say whatever I wanted to say, now I don’t speak a thing to other people.” (Cognitive)

In contrast to the high level of internalized stigma expressed by his mother, IF-E reported a low ISMI score of 1.23 and did not express any thoughts or sentiments about being stigmatized during the semi-structured interview. IF-E believed that no one in his social circle had any particular interest in knowing about his mental illness and emphasized that he did not notice any change in the behavior of other people even if they know.

Finally, dyad-G is a daughter-mother dyad that had returned to the study community 13 years ago after living in an autonomous region in northwest China for nearly 30 years. IP-G, aged 42, had separated from her husband and was living with her 20-year old daughter. FC-G, aged 80 and the third member of the household, had retired with financial support from a pension. While IP-G had been experiencing psychotic symptoms before she moved to the study area, she received a formal diagnosis of schizophrenia only 2 years ago from a mental health professional. IP-G was employed at the time of interview and had worked in the past despite her psychiatric symptoms. FC-G emphasized that IP-G could pass as a “normal” person. Both daughter and mother concurred about keeping mental illness a secret as crucial for the financial prospect of the family. They both spoke about being socially disengaged from their neighbors and coworkers.

“I don’t like talking much and I’m afraid of speaking up. I’m fearful that someone might know. Sometimes I thought I am different from others and I can feel it… Like other people are very cheerful and say many things…I think if it was me, I wouldn’t say those things.” (IP)

“I always hide her illness from others, afraid of others would gossip. I always hide, you see how many years I have been doing this … She was not really that seriously ill. Others cannot tell that she has mental illness.” (FC)

## Discussion

In China, more than three decades of rapid marketization and economic growth has been accompanied by shifting demands on its citizens, leading to elevated levels of psychological distress and anxiety, increasing prevalence of mental disorders, and alarmingly high rates of suicide [40]. Amidst this unprecedented development, the mental health system in China is facing a multitude of challenges, including shortage of a qualified and professional workforce to meet the increasing mental health needs of the Chinese population, immense resources disparities between rural and urban communities, and lack of culturally-informed community treatment approaches [41, 42]. The promulgation of the National Mental Health Law in 2013 is a signpost in community mental health services because of the legislation’s requirement that psychiatric treatment be voluntary in the majority of circumstances [43, 44]. Given that an overwhelming majority of community-dwelling patients with schizophrenia are living with their natural family [23, 27, 45], family is an integral and indispensable component of community mental health services in China. Under the 1981 Chinese Marriage Law, families bear the legal responsibility of the care and management of patients living in the community and they are accountable for the behavior of their ill members [44, 46]. Understanding the experience of stigma and discrimination from both persons with schizophrenia and their family caregivers, therefore, is critical for developing the requisite knowledge and skills base for engaging families affected by mental illness as key stakeholders of the emerging service delivery system [43].

Our findings suggest that perceiving other people as holding devaluating attitudes and behaving discriminatorily was a commonly shared narrative among persons with schizophrenia. Examples abound with regard to various types of maltreatment participants endured, and the circumstances upon which discrimination and devaluation happened. In response to experiencing overt discrimination from employers, co-workers, neighbors and kin, participants spoke about imposing self-restriction on social interactions and community activities for fear of being considered as looking or behaving “weird” by other people. Some participants spoke about the conscious efforts they made to avoid people whom they were familiar with because being around people who did not experience mental illness made them feel out of place or inadequate.

Participants with schizophrenia also expressed a sense of shame and inferiority, spoke about being a burden to their family, and expressed self-disappointment. Interestingly, although there is a moderate level of agreement on items in the stereotype endorsement domain of the ISMI, only one study participant talked about needing others to make decisions on her behalf during the semi-structured interview. The lack of narratives about stereotype endorsement raises question about the degree to which stereotype awareness and self-concurrence, posited as a core construct of self-stigma [3], is relevant for understanding self-stigmatization in Chinese culture. It might be that the word “stereotype” remains an academic term in the Chinese language and has not entered into the everyday lexicon of Chinese citizens. Alternatively, participants in the current study might be living in a close and protective family environment, which prevented them from exposing to severe community censure.

Caregivers spoke profusely about the emotional distress they experienced because of having a family member with mental illness. Emotional responses expressed include “helplessness,” “bitterness,” “profound sadness,” and “worrying for the future.” Seven out of 8 caregivers in the current study were older parents co-residing with their adult children with schizophrenia. Existing literature suggests that older caregivers tend to report higher levels of stress compared to their younger counterparts [47] as they might be apprehensive about the prospect of future caregiving should they lose the capacity to care for their ill relatives when they reach an advanced age. As emotional distress could amplify the practical stresses associated with caregiving, supportive services to address the affective aspect of affiliate stigma needs to be incorporated as a core element of family-based intervention in China [48].

Consistent with the findings from participants with schizophrenia, narratives characterizing perceived discrimination and reduced social engagement are two important dimensions of internalized stigma experienced by family caregivers. Although no caregivers in the semi-structured interviews spoke directly about their reputation being tarnished because of mental illness in the family (a scale item in the Affiliate Stigma Scale), they depicted in detail a variety of situations in which they were looked down upon or ostracized socially. Caregivers generally regarded themselves as being inferior and lesser than others in their social standing, and used avoidance and other strategies to keep mental illness a secret from their relations.

The findings offered in this study can be viewed in the context of a growing body of literature on the cultural ramifications of mental health stigma in Chinese societies. Specifically, the theoretical and empirical work of Yang and colleagues has focused on the pervasive and damaging nature of stigma against mental illness in Chinese culture because of the centrality of “face” [4, 49, 50]. Our study provides a glimpse into the reality that a family’s social standing may be diminished due to a loss of holding onto the cultural expectation of intrapersonal and social-transactional obligations, which when compounded with “loss of face,” leads to social disengagement of family as a social unit.

Our analysis suggests that richer and more nuanced data on the experience of internalized stigma could be generated by adopting a mixed-methods approach integrating quantitative and qualitative data. Although this study is constrained by its small sample size, the data analytic method could be meaningfully extended to larger study samples to guide the development of measurement instruments on stigma that are culturally and linguistically appropriate to Chinese societies. In addition, the analysis of the interactive aspects of stigma in a family dyadic relationship provides an analytical tool for understanding how stigma works within a family unit. This understanding is important given the central role of family in Chinese culture, which vastly influences the help-seeking behavior and treatment adherence in persons living with schizophrenia [8, 51].

A number of limitations are acknowledged for this study. Our sample is limited to 8 family-dyads recruited through a local branch of a national organization that promotes the rights of persons with disabilities. Given the small sample size, the extent to which data from this particular sample may reflect the real situation in the study community is unclear. In addition, all 8 participants with schizophrenia were receiving treatment in the form of psychiatric medications at the time of the interview. In China, the majority of persons with mental illness remains untreated, and even for those that are in the treatment system, most have poor medication compliance [52, 53]. It is, therefore, not clear whether our findings could reflect the lived experiences of such populations.

Response bias is another concern because the participating families were selected out of a potential sample of 30 families. Nevertheless, the study sample allowed us to explore an important phenomenon in a hard to engage population. Small sample sizes are justified when the experience under exploration is specific (narrowly defined), when the community is small, or when the community is extremely hard to engage [54–57].

### Implications

Phillips and colleagues asserted that the internalization of stigma is most damaging to the lives of persons with mental illness and their caregivers but such process can be combated [58]. As a potent barrier for help-seeking, internalized stigma plays a part in contributing to the high prevalence of untreated mental illness in China, which was estimated to be 158 million people, or 91.8% of all individuals with any diagnosis of mental disorders never seeking help [25, 41, 42]. Addressing stigma related to schizophrenia, therefore, can have a broad impact on increasing the treatment rates of other forms of mental illness because schizophrenia epitomizes the most severe form of mental disorder with its “sufferers” being considered by the general public to be dangerous, unpredictable, not fully cultivated, and not competent to participate in social life in China [49]. However, although there has been a proliferation of stigma reduction programs in European Union countries [59] and English-speaking countries [60], a policy agenda for eliminating stigma and promoting public awareness of mental illness is currently lacking in China.

A growing body of studies has shown that positive and direct personal contact with persons with mental illness can serve as an effective anti-stigma strategy for promoting social acceptance [61–64]. Hearing first-voice stories from persons living with mental illness is identified as a key ingredient in stigma reduction programs by allowing program participants to see the person behind the illness, thereby creating empathy and understanding [65]. Narratives by persons living with schizophrenia and their family caregivers documented in the current study provide powerful testimonies on how stigma operates in the context of Chinese culture. And given the reality of social rejection and stigma, the opportunity to speak candidly and authentically about their experience of living in the community with a spoilt identity entails a process of empowering persons with schizophrenia and their family caregivers in believing in their right of community membership, which is consistent with the spirit of the equal opportunity legislation in China [43].

## Conclusion

This study is unique in capturing both the perspectives of persons living with schizophrenia and their family caregivers through matching narratives with standardized scale items, thereby enabling an enriched and contextualized understanding of internalized stigma in a transitioning Chinese community. Despite its exploratory nature as a pilot project, this study contributes to an emerging literature that explores narratives spoken by persons with mental illness and family members to explain the process of how stigma works in undermining social inclusion [29, 30, 66–72]. The study approach adopted in this study could be applied to larger and more representative samples in order to develop culturally-informed measurement tools to capture the experience of internalized stigma as a family unit.

## Additional file

**Additional file 1.** Chinese version of the Internalized Stigma of Mental Illness (ISMI) Scale and the Affiliate Stigma Scale (ASS).
