# Supplementary material for: “My bitterness is deeper than the ocean”: understanding internalized stigma from the perspectives of persons with schizophrenia and their family caregivers
Source: Int J Ment Health Syst. 2018 Apr 3;12:14. doi: 10.1186/s13033-018-0192-4 (PMC5883360; doi:10.1186/s13033-018-0192-4)
Supplement: Supplementary file 1 — Additional file 1. Chinese version of the Internalized Stigma of Mental Illness (ISMI) Scale and the Affiliate Stigma Scale (ASS). [file 13033_2018_192_MOESM1_ESM.docx]

**Chinese version of the Internalized Stigma of Mental Illness (ISMI)**

**心理疾病内化污名量表**

|  | **完全**  **不赞同** | **比较**  **不赞同** | **比较**  **赞同** | **完全**  **赞同** |
| --- | --- | --- | --- | --- |
| **1.因为有心理疾病，我觉得自己不属于这个世界** | 1 | 2 | 3 | 4 |
| **2.人们因我有心理疾病而歧视我** | 1 | 2 | 3 | 4 |
| **3.我避免接近没有心理疾病的人，免得被人拒绝** | 1 | 2 | 3 | 4 |
| **4.我觉得自己不如那些没有心理疾病的人** | 1 | 2 | 3 | 4 |
| **5.和以前相比，我的社交活动变少了，因为心理疾病会让我的样子或行为很“怪异”** | 1 | 2 | 3 | 4 |
| **6.有心理疾病的人不能过上有意义的生活** | 1 | 2 | 3 | 4 |
| **7.我不过多的谈论自己，因为我不想让自己的心理疾病成为别人的负担** | 1 | 2 | 3 | 4 |
| **8.人们对心理疾病有一些负面的看法，这些看法把我排除在社会之外** | 1 | 2 | 3 | 4 |
| **9.和没有心理疾病的人呆在一起，让我觉得不舒服** | 1 | 2 | 3 | 4 |
| **10.我因为有心理疾病而对自己感到失望** | 1 | 2 | 3 | 4 |
| **11.心理疾病毁了我的生活** | 1 | 2 | 3 | 4 |
| **12.因为有心理疾病，我需要别人代我做大部分决定** | 1 | 2 | 3 | 4 |
| **13.我远离社会交往，为的是不让我的家人或朋友蒙羞** | 1 | 2 | 3 | 4 |
| **14.没有心理疾病的人不可能理解我** | 1 | 2 | 3 | 4 |
| **15.只是因为我有心理疾病，人们就不把我当回事** | 1 | 2 | 3 | 4 |
| **16.因为有心理疾病，所以我无法对社会有任何贡献** | 1 | 2 | 3 | 4 |
| **17.因为我有心理疾病，没有人愿意接近我** | 1 | 2 | 3 | 4 |
| **18.因为我有心理疾病，别人认为我在生活中不能取得什么成就** | 1 | 2 | 3 | 4 |
| **19.因为有心理疾病，我成了家人的负担** | 1 | 2 | 3 | 4 |
| **20.我怕谈论和心理疾病相关的话题** | 1 | 2 | 3 | 4 |
| **21.因为有心理疾病，我常常想避开熟悉的人和环境** | 1 | 2 | 3 | 4 |
| **22.我因自己得了心理疾病而讨厌自己** | 1 | 2 | 3 | 4 |

**Chinese Version of Affiliate Stigma Scale (ASS)**

**亲属污名量表**

|  | **完全不赞同** | **比较不赞同** | **比较赞同** | **完全赞同** |
| --- | --- | --- | --- | --- |
| 1. **因为我的家人有精神疾病，所以我感到自卑。** | 1 | 2 | 3 | 4 |
| 1. **我避免与有精神疾病的家人沟通。** | 1 | 2 | 3 | 4 |
| 1. **别人会因为我与有精神疾病的家人在一起而歧视我。** | 1 | 2 | 3 | 4 |
| 1. **因为我的家人有精神疾病，所以我有情绪上的困扰。** | 1 | 2 | 3 | 4 |
| 1. **我不敢向外人透露我家里有精神疾病患者。** | 1 | 2 | 3 | 4 |
| 1. **因为我的家人有精神疾病，所以我的声誉蒙受损害。** | 1 | 2 | 3 | 4 |
| 1. **有精神疾病的家人的行为令我感到困窘。** | 1 | 2 | 3 | 4 |
| 1. **我会减少与有精神疾病的家人一起外出。** | 1 | 2 | 3 | 4 |
| 1. **当我和有精神疾病的家人在一起时，别人对我的态度会变差。** | 1 | 2 | 3 | 4 |
| 1. **因为我的家人有精神疾病，所以我觉得无助。** | 1 | 2 | 3 | 4 |
| 1. **因为我的家人有精神疾病，所以我减少与亲戚朋友联络。** | 1 | 2 | 3 | 4 |
| 1. **「我的家人有精神疾病」这件事对我有负面影响。** | 1 | 2 | 3 | 4 |
| 1. **因为我的家人有精神疾病，所以我感到伤心。** | 1 | 2 | 3 | 4 |
| 1. **当我与有精神疾病的家人在一起时，我会比较低调。** | 1 | 2 | 3 | 4 |
| 1. **「我的家人有精神疾病」这件事令我觉得自己比不上别人。** | 1 | 2 | 3 | 4 |
| 1. **我担心别人会知道我的家人有精神疾病。** | 1 | 2 | 3 | 4 |
| 1. **我减少与有精神疾病的家人来往。** | 1 | 2 | 3 | 4 |
| 1. **因为我的家人有精神疾病，所以我觉得自己低人一等。** | 1 | 2 | 3 | 4 |
| 1. **因为有家人是精神疾病患者，所以我觉得压力很大。** | 1 | 2 | 3 | 4 |
| 1. **我不敢参与和精神疾病有关的活动，免得别人怀疑我的家人有精神疾病。** | 1 | 2 | 3 | 4 |
| 1. **「我的家人有精神疾病」这件事令我觉得丢脸。** | 1 | 2 | 3 | 4 |
| 1. **因为我的家人有精神疾病，所以我减少与邻居接触。** | 1 | 2 | 3 | 4 |
